# COVID-19 and autoinflammatory diseases: prevalence and outcomes of infection and early experience of vaccination in patients on biologics

**DOI:** 10.1093/rap/rkab043

**Published:** 2021-08-23

**Authors:** Claire J Peet, Charalampia Papadopoulou, Bella Ruth M Sombrito, Michael R Wood, Helen J Lachmann

**Affiliations:** 1 CAPS and Autoinflammatory Diseases Treatment Service, National Amyloidosis Centre, Division of Medicine, University College London, Royal Free Hospital; 2 Department of Medical and Molecular Genetics, King’s College London, London, UK

**Keywords:** systemic autoinflammatory diseases, COVID-19, vaccination, adenoviral vector, mRNA vaccine, biologics

## Abstract

**Objectives:**

The systemic autoinflammatory diseases are rare conditions; to date, data on coronavirus disease 2019 (COVID-19) infection and vaccination safety are scarce. Agents targeting innate immune pathways have transformed the management of affected patients, and their outcomes are of wider interest given the role of inflammation in both viral clearance and severe COVID-19 disease. We surveyed patients with systemic autoinflammatory disease on biologic therapy to determine the prevalence and outcomes of COVID-19 infection and to gather early safety data on vaccination.

**Methods:**

Electronic medical records of 248 patients with systemic autoinflammatory disease on biologic therapy at a national centre were reviewed. Patients were then surveyed in clinic or using a Web-based survey.

**Results:**

In the cohort of 248 patients, no deaths were recorded. One hundred and seventy-five survey responses were received. Among the respondents, 27 reported suspected COVID-19 infection, of which 14 were confirmed by testing (8.0%). Two patients required hospital admission owing to dehydration. No patient required respiratory support or intensive care. One hundred and thirty-eight doses of COVID-19 vaccine had been administered to 130 patients. Side effects were reported after 71 of 138 (51.4%) administrations and were consistent with a flare of the underlying disease in 26 of 138 (18.8%) instances. No serious adverse events or hospital admissions were reported after vaccination.

**Conclusion:**

These data, including the largest published series of patients on anti-IL-1/6 biologics to receive any adenoviral vector or messenger RNA vaccine, show no serious early concerns regarding vaccination and will provide an urgently needed resource to inform decision-making of these patients and their clinicians.

**Key messages**

- Coronavirus disease 2019 (COVID-19) infection occurred in patients with systemic autoinflammatory diseases at population expected rates and was generally uncomplicated.
- COVID-19 vaccination was generally well tolerated in this series, with no serious adverse events reported.
- This includes the largest report of patients on anti-IL-1/6 biologics receiving any adenoviral vector or mRNA vaccine.

## Introduction

A recurring issue raised by the coronavirus disease 2019 (COVID-19) pandemic is the role of inflammatory cytokines in the balance between viral clearance and hyper-inflammation mediating severe disease. Cytokine-modulating agents have been assessed in clinical trials in COVID-19 infection, and IL-6 (but not IL-1) blockade is now part of the standard care of patients requiring respiratory support [1]. The systemic autoinflammatory diseases (SAIDs) are the paradigm of cytokine-driven disorders of innate immunity and, over the last 25 years, research into their pathophysiology has provided key insights into the mechanisms underlying systemic inflammation [2]. Consequently, outcomes in these patients and, in particular, those on biologics that inhibit key inflammatory pathways, such as IL-1, IL-6 and TNF-α, might provide helpful insights into the pathophysiology of this new disease.

For patients with these conditions and their clinicians, there is currently an almost complete absence of data to guide decision-making on COVID-19 vaccination and on continuation of biologic therapy where risk of COVID-19 infection is high or proven. Problematically, pre-pandemic consensus guidelines and biologic drug product information do not contain guidance pertaining to the classes of vaccine, mRNA and non-replicating adenoviral vector, currently approved for use for COVID-19 [3, 4]. More recent EULAR recommendations advocate the use of any approved COVID-19 vaccine in patients with rheumatic diseases on biologics, including patients with SAID, although no data specifically pertaining to their safety in this patient group have been published [5]. Additionally, reports of adverse reactions to certain vaccinations in patients with SAID have contributed to uncertainty and anxiety around the take-up of the vaccination in this population [6, 7]. To begin to address these areas, here we describe our experience of COVID-19 over the first year of the pandemic and our early data on vaccination in a UK cohort of adult patients with SAID on long-term cytokine-modulating therapy.

## Methods

Electronic medical records of 248 patients with SAID on biologic therapy and under active follow-up at a national centre for management of autoinflammatory diseases were reviewed for diagnosis, treatment, hospital admission data and mortality data. These patients were then surveyed on experiences of COVID-19 infection and vaccination using a Web-based survey that was completed by the patient or their carer, or by their clinician during a clinic appointment, between 4 March 2021 and 26 March 2021 inclusive. Data analysis and visualization were performed in R statistics (v.4.0.3; https://www.R-project.org). Informed written consent was provided by all patients, and the study was conducted in accordance with ethical approval from the Royal Free Hospital and University College Medical School Research Ethics Committee (REC 06/Q0501/42).

## Results

**Fig. 1:**
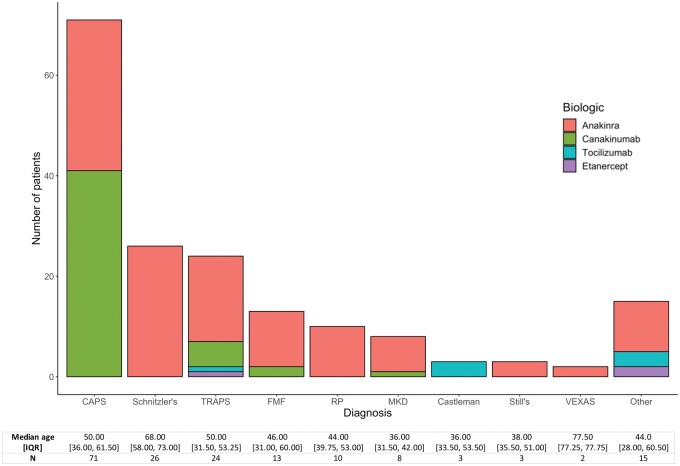
Patient characteristics of survey respondents Diagnosis and therapeutic agents of 175 patients with SAID on biologic therapy who responded to a survey on coronavirus disease 2019 infection and vaccination. CAPS: cryopyrin-associated periodic syndrome; E1 enzyme, X-linked autoinflammatory somatic syndrome; MKD: mevalonate kinase deficiency; RP: recurrent pericarditis; SAID: systemic autoinflammatory disease; TRAPS: TNF receptor-associated periodic syndrome; VEXAS: vacuoles.

In the cohort of 248 patients, no deaths were recorded between 29 January 2020 and 26 March 2021. Survey responses were received from 175 of 248 patients (70.6%). Diagnoses and biologic therapy of respondents are summarized in Fig. 1. Additional data on demographics, co-morbidity and treatment are summarized in Supplementary Table S1, available at *Rheumatology Advances in Practice* online. Over this first year of the pandemic, all patients were advised, in line with national guidance, that autoinflammatory disease with single-line biologic treatment was not an indication for shielding and that they should not discontinue their medication. Twenty-seven of 175 (15.4%) patients reported suspected COVID-19 infection, of which 14 cases were confirmed by laboratory testing (8.0%), including one asymptomatic infection detected by surveillance screening. No patients required supplemental oxygen, ventilatory support or intensive care unit admission. Two patients on anakinra required hospital admission because of vomiting and dehydration. Anakinra was discontinued temporarily in the two patients admitted to hospital by the admitting teams. All patients with confirmed COVID-19 infection who did not require hospital admission continued their biologic therapy without interruption. Two patients reported persistent, otherwise unexplained symptoms >12 weeks after COVID-19 infection, consistent with a diagnosis of post-COVID syndrome as currently defined [8].

One hundred and thirty of 175 (74.3%) patients had received a first vaccine, and eight had received the second dose in line with national guidance on dose intervals. The characteristics of vaccinated patients and reported vaccine reactions are summarized in Table 1. Two patients only, on anakinra and tocilizumab, respectively, suspended therapy for two days around vaccination. All other patients continued their biologic therapy uninterrupted. Side effects were reported after 71 of 138 (51.4%) vaccine administrations. After 26 of 138 (18.8%) administrations, patients reported systemic symptoms in keeping with their underlying SAID. No patient required hospital admission after vaccination, and no thromboembolic events or other serious adverse events were reported.

**Table 1:** Patient characteristics and vaccine reactions by coronavirus disease 2019 vaccine dose administered

	Oxford-AstraZeneca (*n* = 74)	Pfizer-BioNTech (*n* = 64)
**Patient characteristics**		
Age, median [IQR], years	56.00 [38.00–66.75]	56.00 [42.75–67.00]
Diagnosis, *n* (%)		
CAPS	29 (39.2)	22 (34.4)
Schnitzler’s syndrome	14 (18.9)	10 (15.6)
TRAPS	11 (14.9)	8 (12.5)
Recurrent pericarditis	3 (4.1)	8 (12.5)
FMF	2 (2.7)	6 (9.4)
MKD	3 (4.1)	3 (4.8)
Adult-onset Still’s disease	2 (2.7)	0 (0.0)
Castleman disease	1 (1.4)	0 (0.0)
VEXAS syndrome	1 (1.4)	1 (1.6)
Other	8 (10.8)	6 (9.5)
Biologic, *n* (%)		
Anakinra	47 (63.5)	44 (68.8)
Canakinumab	23 (31.1)	14 (21.9)
Tocilizumab	4 (5.4)	2 (3.1)
Etanercept	0 (0.0)	4 (6.2)
**Vaccine dose administered**		
First dose, *n* (%)	73 (98.6)	57 (89.1)
Second dose, *n* (%)	1 (1.4)	7 (10.9)
**Side effects**		
Side effects reported, *n* (%)	43 (58.1)	28 (43.8)
Localized symptoms, *n* (%)	26 (36.6)	18 (28.6)
Systemic symptoms, *n* (%)	41 (57.7)	26 (41.3)
Fatigue, *n* (%)	30 (42.3)	17 (27.0)
Myalgia, *n* (%)	20 (28.2)	15 (23.8)
Fever, *n* (%)	15 (21.1)	5 (7.9)
Headache, *n* (%)	8 (11.3)	5 (7.9)
Consistent with SAID flare, *n* (%)	20 (27.0)	6 (9.4)
Time off work required, *n* (%)	21 (28.4)	12 (18.8)
Median [IQR] days	2.0 [1.0–3.0]	2.0 [1.0–2.0]
**Serious adverse events**		
Serious adverse events, *n* (%)	0 (0.0)	0 (0.0)
Hospital admissions, *n* (%)	0 (0.0)	0 (0.0)
Deaths, *n* (%)	0 (0.0)	0 (0.0)

CAPS: cryopyrin-associated periodic syndrome; E1 enzyme, X-linked autoinflammatory somatic syndrome; IQR: interquartile range; MKD: mevalonate kinase deficiency; SAID: systemic autoinflammatory disease; TRAPS: TNF receptor-associated periodic syndrome; VEXAS: vacuoles.

## Discussion

The 8.0% prevalence of confirmed COVID-19 infection in our series of patients with SAID on biologic therapy is broadly in line with that reported for the general UK adult population. Compared with the reported Parisian experience of familial Mediterranean fever, our patients have had relatively mild disease, with no deaths or requirement for respiratory support [9]. All patients with COVID-19 managed in the outpatient setting continued their biologics without adverse events, consistent with a previous smaller published series of patients on anakinra [10]. Taken together, these data support our current practice of continuing biologic therapy in this group when COVID-19 is prevalent in the general population and during uncomplicated COVID-19 infection.

As the vaccination programme proceeds, there is an urgent need for real-world clinical data to facilitate informed decision-making on the part of clinicians and patients with SAID. Recent EULAR guidance recommend the use of any approved COVID-19 vaccine in these patients, although safety and efficacy data pertaining specifically to patients with SAID or on biologics are yet to be published [5]. In our series, vaccination has been offered in line with this guidance and thus far has been well tolerated, with no serious adverse events and side effects consistent with those reported in trials [11, 12].

The study has a number of limitations. This is a real-world observational study with a short follow-up time after vaccination and, given that SAIDs are rare, the number of COVID-19 infections reported is small. Additionally, although our survey response rate was high (70.6%), we cannot exclude a non-response bias, which would be expected to cause over-estimation of rates of COVID-19 infection and vaccine reactions. Finally, a key question remains regarding vaccine the efficacy of COVID-19 vaccination and, as such, there is a need for further studies investigating the degree of humoral and cellular response to vaccination in this patient group.

Nonetheless, there is a pressing need for timely sharing of data sets like this in a global pandemic, particularly in a rare patient population where there are legitimate concerns owing to historical reports of vaccine reactions and inconsistent guidance [3–7]. Moreover, this is the largest series reported of the use of any viral vector or mRNA vaccine in patients on anti-IL-1/6 agents. It therefore provides much needed real-world data to support newly issued guidance advocating the use of these novel classes of vaccine in these patients [3, 4]. Although vaccine efficacy in these patients remains the key outstanding question, these preliminary safety data provide a useful reassurance that will inform clinician and patient decision-making worldwide.

## Supplementary Material

rkab043_Supplementary_Data
