# Supplementary material for: COVID-19 and autoinflammatory diseases: prevalence and outcomes of infection and early experience of vaccination in patients on biologics
Source: Rheumatol Adv Pract. 2021 Aug 23;5(2):rkab043. doi: 10.1093/rap/rkab043 (PMC8397842; doi:10.1093/rap/rkab043)
Supplement: rkab043_Supplementary_Data [file rkab043_supplementary_data.docx]

**Supplementary Table S1.** Demographics and medical history of survey respondents

|  |  | **All respondents (n=175)** |
| --- | --- | --- |
| **Demographics** | | |
|  | Age, median [IQR] | 50.00 [37.00 -64.00] |
|  | Sex, female (%) | 95 (64.3) |
|  | Ethnicity, n (%) |  |
|  | White or White British | 155 (88.6) |
|  | Asian or Asian British | 10 (5.7) |
|  | Black or Black British | 3 (1.7) |
|  | Other ethnic background | 7 (4.0) |
|  | UK region of residence, n (%) |  |
|  | South East | 52 (29.7) |
|  | Greater London | 28 (21.7) |
|  | East of England | 19 (10.9) |
|  | West Midlands | 16 (9.1) |
|  | North West England | 15 (8.6) |
|  | South West England | 13 (7.4) |
|  | East Midlands | 5 (2.9) |
|  | Yorkshire and Humber | 5 (2.9) |
|  | Northern Ireland | 3 (1.7) |
|  | Wales | 3 (1.7) |
|  | Scotland | 2 (1.1) |
| **Diagnoses** | | |
|  | Autoinflammatory disease diagnosis, n (%) |  |
|  | CAPS | 71 (40.6) |
|  | Schnitzler’s syndrome | 26 (14.9) |
|  | TRAPS | 24 (13.7) |
|  | Recurrent pericarditis | 10 (5.7) |
|  | FMF | 13 (7.4) |
|  | MKD | 8 (4.6) |
|  | Adult onset Still’s disease | 3 (1.7) |
|  | Castleman disease | 3 (1.7) |
|  | VEXAS syndrome | 2 (1.1) |
|  | Other | 15 (8.6) |
|  | Other co-morbidities, n (%) |  |
|  | Hypertension | 30 (17.1) |
|  | Underlying respiratory disease | 17 (9.7) |
|  | Chronic kidney disease | 9 (5.1) |
|  | Ischaemic heart disease | 9 (5.1) |
|  | Malignancy (exc. localised skin cancer) | 9 (5.1) |
|  | Diabetes mellitus | 7 (4.0) |
|  | Previous venothromboembolic event(s)^1^ | 3 (1.7) |
|  | Other neurological disease^2^ | 2 (1.1) |
|  | None of the above | 115 (65.7) |
| **Immunomodulatory therapy** | | |
|  | Biologic, n (%) |  |
|  | Anakinra | 116 (66.3) |
|  | Canakinumab | 49 (28.0) |
|  | Tocilizumab | 7 (4.0) |
|  | Etanercept | 3 (1.7) |
|  | Other immunomodulatory therapy, n (%) |  |
|  | No other immunomodulatory therapy | 142 (81.1) |
|  | Colchicine | 18 (10.3) |
|  | Corticosteroids | 15 (8.6) |
|  | Other immunosuppressant therapy | 8 (4.6) |
| *CAPS: cryopyrin associated periodic syndrome; TRAPS: TNF receptor associated periodic syndrome; FMF: familial Mediterranean fever; MKD: mevalonate kinase deficiency; VEXAS: vacuoles, E1 enzyme, X linked autoinflammatory somatic syndrome; SAID: systemic autoinflammatory disease. ^1^No patients had a history of thrombosis with thrombocytopenia, or cerebral venous sinus thrombosis ^2^Other neurological disease does not include patients with neurological involvement resulting from their autoinflammatory disease, and refers instead to other neurological co-morbidities, such as previous stroke, multiple sclerosis, Parkinson’s disease et.* | | |
